# Design, Synthesis, and *In Vitro* Kinetics Study of Atenolol Prodrugs for the Use in Aqueous Formulations

**DOI:** 10.1155/2014/248651

**Published:** 2014-01-12

**Authors:** Rafik Karaman, Alaa Qtait, Khulod Khayyat Dajani, Saleh Abu Lafi

**Affiliations:** ^1^Faculty of Pharmacy, Al-Quds University, P.O. Box 20002, Jerusalem, Palestine; ^2^Department of Sciences, University of Basilicata, Via dell'Ateneo Lucano 10, 85100 Potenza, Italy; ^3^Faculty of Public Health Sciences, Al-Quds University, P.O. Box 20002, Jerusalem, Palestine

## Abstract

Based on DFT, MP2, and the density functional from Truhlar group (hybrid GGA: MPW1k) calculations for an acid-catalyzed hydrolysis of nine Kirby's N-alkylmaleamic acids and two atenolol prodrugs were designed. The calculations demonstrated that the amide bond cleavage is due to intramolecular nucleophilic catalysis by the adjacent carboxylic acid group and the rate-limiting step is determined based on the nature of the amine leaving group. In addition, a linear correlation of the calculated and experimental rate values has drawn credible basis for designing atenolol prodrugs that are bitterless, are stable in neutral aqueous solutions, and have the potential to release the parent drug in a sustained release manner. For example, based on the calculated B3LYP/6-31 G (d,p) rates, the predicted *t*_1/2_ (a time needed for 50% of the prodrug to be converted into drug) values for atenolol prodrugs ProD 1-ProD 2 at pH 2 were 65.3 hours (6.3 hours as calculated by GGA: MPW1K) and 11.8 minutes, respectively. *In vitro* kinetic study of atenolol prodrug ProD 1 demonstrated that the *t*_1/2_ was largely affected by the pH of the medium. The determined *t*_1/2_ values in 1N HCl, buffer pH 2, and buffer pH 5 were 2.53, 3.82, and 133 hours, respectively.

## 1. Introduction

Atenolol, 4-[2-hydroxy-3-[(1-methylethyl)amino]propoxy] benzene acetamide, is a relatively polar hydrophilic compound with a water solubility of 26.5 mg/mL at 37°C and a log partition coefficient (octanol/water) of 0.23. Lipid insoluble hydrophilic compounds such as atenolol, sotalol, and nadolol are excreted only by the kidneys and have low brain penetration. Atenolol is a selective *β*_1_-adrenoceptor antagonist, applied in the treatment of numerous cardiovascular disorders including hypertension, angina, acute myocardial infarction, supraventricular tachycardia, ventricular tachycardia, and the symptoms of alcohol withdrawal *via* restricting certain nerve impulses, thereby controlling the rate and force of contraction and consequently reducing blood pressure in addition to its treatment of angina pectoris. On the outset, atenolol actively reduces the heart rate by decreasing systolic and diastolic blood pressure. The net effect of atenolol on controlling both the heart rate and blood pressure is the reduction in myocardial work and oxygen requirement which reduces cardiovascular stress, thereby preventing arrhythmia and angina attacks.

Atenolol is marketed as tablets of 25 mg, 50 mg, and 100 mg and an injectable formulation of 5 mg/mL supplied in 10 mL ampoules [1, 2]. Atenolol has a p*K*_a_ of 9.6; it undergoes ionization in the stomach and intestine, thus its oral bioavailability is low due to inefficient absorption through membranes. The bioavailability of atenolol is between 45% and 55% of the given dose and is not increased by administration of the drug in a solution form [3–5]. About 50% of administered atenolol is absorbed; however, most of the absorbed quantity reaches the systemic circulation. Atenolol peak blood levels are reached within two to four hours after ingestion. Differently from propranolol or metoprolol, atenolol is resistant to metabolism by the liver and the absorbed dose is eliminated by renal excretion. More than 85% of IV dose is excreted in urine within 24 hours compared with 50% for an oral dose. Only 6–16% is protein bound resulting in relatively consistent plasma drug levels with about a fourfold interpatient variation. The elimination half-life of atenolol is between 6 and 7 hours and there is no alteration of kinetic profile of a drug by chronic administration. Atenolol is one of the most important medicines used for prevention of several types of arrhythmias in childhood, but unfortunately it is still unlicensed [6]. On the other hand, atenolol is indicated as a first-step therapy for hypertension in elderly patients, who have difficulty in swallowing and, thus, tablets and capsules are frequently avoided. The ease of administration makes a liquid formulation an ideal dosage form for such patients [7]. Therefore, extemporaneous compounding (off-label), involving preparation of an oral liquid from a pure drug powder, is required. However, formulations compounded from tablets and pure active drug suffer instability and are only stable for less than one week [8–10]. Furthermore, atenolol bitterness is considered as a great challenge to health sector when used among children and geriatrics [11]. The main problem in oral administration of bitter drugs such as atenolol is incompliance by the patients [12] and this can be overcome by masking the bitterness of a drug either by decreasing its oral solubility on ingestion or eliminating the interaction of drug particles to taste buds [13].

There are various techniques available which are commonly used for masking drug's bitterness: (1) taste masking with flavors, sweeteners, and amino acids [14]; (2) taste masking with lipophilic vehicles such as lipids, lecithin, and lecithin-like substances [15]; (3) coating which is classified based on the type of coating material, coating solvent system, and the number of coating layers [16]; (4) microencapsulation based on the principle of solvent extraction or evaporation [17]; (5) sweeteners are generally used in combination with other taste masking technologies [18]; (6) taste suppressants and potentiators, such as Linguagen's bitter blockers (e.g., adenosine monophosphate), are used for masking the bitter taste of various compounds by competing with binding to the G-protein coupled receptor sites (GPCR) [19]; (7) resins are utilized to mask pharmaceuticals bitterness by forming insoluble resonates [20, 21]; (8) inclusion complex by which the drug molecule fits into the cavity of a complexing agent and forms a stable complex that masks the bitter taste of a drug by decreasing its oral solubility [22]; (9) pH modifiers are capable of generating a specific pH microenvironment in aqueous media that has the ability to facilitate *in situ* precipitation of the bitter drug compound in saliva thus reducing the overall taste sensation for liquid dosage forms [23]; (10) adsorbates, by which the compound may be adsorbed or entrapped in the matrix of the adsorbate pore, which may result in a delayed release of the bitter tastant during passage through the oral cavity and mask the taste [24]; and (11) prodrug approach by which the functional group/s bind to the bitter taste receptor is blocked by a promoiety [25].

Previous studies on stability of atenolol ester prodrugs for the use in transdermal preparations have shown that these ester derivatives are much more stable than the corresponding alcohol, atenolol, when they are formulated in aqueous solutions [26, 27]. On the other hand, the only atenolol prodrug intended to be used for oral dosage form was atenolol aspirinate prodrug (aspirin); it is described for antihypertensive therapy to reduce cardiovascular death, stroke, and myocardial infarction (MI); however, recent studies reported that the coupling of atenolol with acetyl salicylic acid by means of an ester linkage did not produce any efficient pharmacological profile, neither *in vitro* nor *in vivo *[28].

Many therapeutic drugs have undesirable properties that may become pharmacological, pharmaceutical, or pharmacokinetic barriers in clinical drug application; one of the most promising strategies for precise and efficient drug delivery and enhancement of a drug therapeutic effect is the prodrug design approach which is becoming more elaborate in the development of efficient and selective drug delivery systems [29]. Approximately, tenth of all worldwide marketed medicines can be categorized as prodrugs, and, in 2008 alone, third of all approved small-molecular-weight drugs were prodrug [30]. This data signifies the importance of the prodrug approach and encourages the scientific community to consider it in the early stages of preclinical development and not as a last resort [31].

Prodrugs are substances administered in an inactive form that is then metabolized in the body into the corresponding active drug. The rationale behind administering prodrugs is to optimize the drug absorption, distribution, metabolism, and excretion. Since first described in the 1950s, prodrugs continue to be a fertile area of research. There are a number of small pharmaceutical/biotech companies dedicated to use prodrugs for the delivery of older but problematic drugs as well as to develop broad-based prodrug technologies for application to new and future drugs [32].

The use of the term prodrug usually implies a covalent link between a drug and a chemical promoiety. Generally, prodrugs can be enzymatically or nonenzymatically converted *in vivo* to provide the parent active drug which exerts the desired therapeutic effect. Ideally, the prodrug should be converted to the parent drug as soon as its goal is achieved, followed by the subsequent rapid elimination of the released promoiety group [33, 34].

The novel prodrug design approach that was utilized for the synthesis and kinetic studies described in this paper is based on intramolecular process using molecular orbital methods and correlations between experimental and calculated values using density functional theory (DFT) [35] and molecular mechanics (MM) [36] methods which are increasingly being exploited and widely recognized as tools for providing structure-energy calculations for predictions of potential drugs and prodrugs [37]. In this approach, no enzyme is needed for the catalysis of the intraconversion of a prodrug to its parent drug. The rate of the drug release is dependent solely on the rate-limiting step of the intraconversion process. Therefore, the reaction mechanism must be investigated in order to assign the factors playing role in the reaction rate. This information is then utilized to design an efficient chemical device to be exploited as a prodrug linker capable of liberating the parent drug in a programmable manner (slow or fast release).

As a result, the intended prodrugs can be designed to undergo cleavage reactions in physiological environments such as stomach (pH 1-2), intestine (pH 5.5-7), and/or blood circulation (pH 7.4), with rates that are solely dependent on the structural features of the pharmacologically inactive promoiety.

Computational chemistry methods based on quantum molecular (QM) and molecular mechanics (MM) theories could be useful for the design of innovative prodrugs for hydroxyl, phenol, or amine containing drugs. For instance, mechanisms of intramolecular processes for a respected number of enzyme models that were previously investigated by others to understand enzyme catalysis have been recently explored by us and exploited for a design of some new novel prodrugs. Using DFT, molecular mechanics, and *ab initio* at different levels, numerous enzyme model processes were calculated for determining the factors governed their rates. These processes include (i) intramolecular acid-catalyzed hydrolysis of N-alkylmaleamic acid derivatives [38, 39]; (ii) proton transfers between two oxygen atoms and between oxygen and nitrogen in Kirby's acetals [40–48]; (iii) proton transfer between two oxygen atoms in nonflexible (rigid) systems as studied by Menger and Ladika [49–53]; (iv) acid-catalyzed ring-closing reactions of hydroxy-acids as investigated by Milstien and Cohen [54–57] and Menger and Ladika [49–53]; and (v) S_N_2-based cyclization reactions as researched by Brown and van Gulick [58], Bruice and Pandit [59, 60], and Galli and Mandolini [61].

These studies have demonstrated that there is a must to further explore the mechanisms for the above-mentioned processes for assigning the factors affecting the nature and the mode of the reaction. Unraveling the reaction mechanism would allow for an accurate design of an efficient chemical device to be utilized as a prodrug promoiety that can be covalently linked to a parent drug to provide chemically and not enzymatically the parent drug in a programmable manner upon exposure to physiological environments. For example, exploring the mechanism for proton transfer in Kirby's acetals has led to design and synthesis of novel prodrugs of aza-nucleosides to treat myelodysplastic syndromes [62], statins to treat high cholesterol blood levels [63], bitterless paracetamol prodrugs to be administered to children and elderly as antipyretic and pain killer [25], and prodrugs of phenylephrine as decongestants [64]. In the above-mentioned examples, the prodrug moiety was attached to the hydroxyl group of the active drug such that the drug promoiety (prodrug) has a potential to degrade upon exposure to physiological environment such as stomach, intestine, and/or blood circulation, with rates that are solely dependent on the structural features of the pharmacologically inactive promoiety (Kirby's enzyme model). Other different linkers such as Kirby's N-alkylmaleamic acids (enzyme model) were also investigated for the design of some prodrugs such as those of tranexamic acid to treat bleeding conditions [65] and acyclovir (antiviral drug) to treat Herpes Simplex [66]. Further, prodrugs for masking the bitterness of antibacterial drugs such as cefuroxime were designed and made as well [67]. The role of the promoiety in the antibacterial (cefuroxime) and paracetamol prodrugs was to block the free amine (cefuroxime) or phenol (paracetamol), which is believed to be responsible for the drug bitterness, and to enable the release of the drug in a programmable manner. Menger's Kemp acid enzyme model was also exploited for the design of dopamine prodrugs for the treatment of Parkinson's disease [68].

The goals of this work were (1) design of atenolol prodrugs that can be (a) formulated in aqueous solutions and maintain stability over a long period of time and (b) bitterless and capable of undergoing intraconversion in physiological environment to provide the parent active drug, atenolol, in a programmable manner and (2) synthesis, characterization, and *in vitro* kinetic study of the interconversion of the designed prodrugs in different media: 1 N HCl and at buffers of pH 2, pH 5, and pH 7.4.

## 2. Materials and Methods

### 2.1. Instrumentations

The melting points were determined in open capillaries on electrothermal stuart SMP3 advanced melting point apparatus; IR spectra were obtained from a KBr matrix (4000–400 cm^−1^) using a Perkin-Elmer, Spectrum 100, FT-IR spectrometer.

The LC/ESI-MS/MS system used was Agilent 1200 series liquid chromatography coupled with a 6520 accurate mass quadruple-time of flight mass spectrometer (Q-TOF LC/MS). The analysis was performed in the positive electrospray ionization mode. The *m/z* range for MS scans was 100–1600 Da. The high pressure liquid chromatography (HPLC) system consisted of an Alliance 2695 module coupled with 2996 Photodiode array detector from Waters (Germany). Data acquisition and control were carried out using Empower 2 software (Waters, Germany). Analytes were separated on a 4.6 mm × 150 mm XBridge C18 column (5 *μ*m particle size) used in conjunction with a 4.6 × 20 mm, XBridge C18 guard column. Microfilters of 0.45 *μ*m porosity were normally used (Acrodisc GHP, Waters).

^1^H-NMR experiments were performed with a Bruker AvanceII 500 spectrometer equipped with a 5 mm BBO probe. pH values were recorded on pH meter model HM-30G: TOA electronics; thin-layer chromatography (TLC) was carried out on TLC plastic sheets silica gel, 20 × 20 cm, layer thickness 0.2 mm, and the spots on the plates were localized by exposure to UV light.

### 2.2. Chemicals and Reagents

Pure atenolol standard was commercially available; maleic anhydride, anhydrous sodium dihydrogen phosphate, sodium hydroxide, concentrated hydrochloric acid, and anhydrous magnesium sulfate were commercially obtained from sigma Aldrich (Israel). HPLC grade solvents of methanol, acetonitrile, and water were purchased from Sigma Aldrich (Israel). High purity chloroform, DMF, and diethyl ether (>99%) were purchased from Biolab (Israel).

### 2.3. Preparation of Atenolol ProD 1

**Figure 1:**
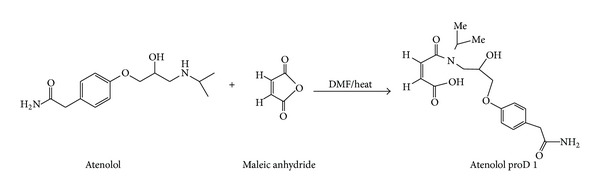
Synthetic scheme for the preparation of atenolol ProD 1 from its parent drug, atenolol and maleic anhydride.

Synthesis of the atenolol ProD 1 was accomplished using Kirby's procedure [38] (Figure 1); in a 250 mL round bottom flask equipped with a stirrer and a condenser, standard atenolol (10 mmol) was dissolved in DMF (10 mL), then maleic anhydride (10 mmol) was added, the reaction mixture was refluxed for four hours and cooled and the DMF was removed by evaporation under vacuum, the resulting residue was dissolved in 20 mL 1 N NaOH, extracted with 3 × 30 mL of ether, the organic layer was dried over anhydrous MgSO_4_, and the dry residue was crystallized (yield 81%). The melting point of the resulting white precipitate was 105°C, and UV (*λ*_max_) was 276 nm. ^1^H NMR *δ* (ppm) (500 MHz, MeOD) 1.16–1.23 (d, 6H, CH_3_CHCH_3_), 3.45–3.47 (d, 2H, *J* = 1.4 Hz, NH_2_-CO-CH_2_–), 3.49-3.50 (m, 2H, N–CH_2_–) 3.52–3.55 (m, 1H, –NCH(CH_3_)_2_), 4.00-4.01 (m, 1H, CH_2_ CH(OH)CH_2_), 4.24–4.27 (m, 1H, –O–CH_2_–), 4.41–4.43 (m, 1H, –OCH_2_–), 5.96 (s, 2H, –NH_2_), 6.04–6.07 (d, 1H, *J* = 13.3 Hz, –CH=CH–), 6.28–6.32 (d, 1H, *J* = 13.3 Hz, –CH=CH–), 6.88–6.93 (m, 2H, aromatic), 7.18–7.22 (m, 2H, aromatic). IR (KBr/*ν*_max_ cm^−1^) 1712 (C=O), 1643 (C=O), 1587 (C=C), 1420, 1280, 1200, 1132, 1058. LC-ESi-MS (positive mode)* m*/*z *(M+1)^+^ found 365.1705 Da, calculated 365.1712 Da.

### 2.4. Kinetic Method

#### 2.4.1. Buffer Preparation

Buffer pH 2 : 6.8 g potassium dihydrogen phosphate were dissolved in 900 mL water for HPLC and the pH was adjusted by diluted *o*-phosphoric acid and water was added to a final volume of 1000 mL (0.05 M). The same procedure was done for the preparation of buffers pH 5 and 7.4; however, in these two cases, the pH was adjusted using 1 N NaOH.

#### 2.4.2. Calibration Curve for Atenolol and Atenolol ProD 1

To construct a calibration curve for atenolol prodrug and the parent drug, atenolol, several concentrations of 10, 50, 100, 200, 300, and 500 ppm were prepared. All samples were injected into HPLC-PDA. The optimal HPLC conditions used for the analysis of atenolol were 4.6 mm × 150 mm, 5 *μ*m, XBridge C18 column, a mobile phase consisting of binary solvent mixture of water: acetonitrile (water pH adjusted to 3.4 using dilute *o*-phosphoric acid) (55 : 45 v/v), a flow rate of 0.5 mL/minute, and a UV detection at a wavelength of 230 nm. Peak area versus concentration of the pharmaceutical (ppm) was then plotted, and the *R* ^2^ value of the plot was recorded.

#### 2.4.3. Preparation of Standard and Sample Solutions

500 ppm of standard atenolol was prepared by dissolving 50 mg of the drug in 100 mL 1 N HCL, buffer pH 3, buffer pH 5, or buffer pH 7.4. Ten *μ*L of the sample was injected into the HPLC to detect the retention time of atenolol. The same procedure was followed for the preparation of 500 ppm of atenolol prodrug.

#### 2.4.4. Hydrolysis of Atenolol ProD 1

Atenolol ProD 1 rate hydrolysis was studied at 37°C in buffer solutions at different pHs (1 N HCl, pH 2, pH 5, and pH 7.4); samples of the reaction mixtures were analyzed directly by HPLC; quantitative analysis of atenolol prodrug and its hydrolysis product, atenolol, was obtained using a calibration curve.

### 2.5. Calculations Methods

The Becke three-parameter, hybrid functional combined with the Lee, Yang, and Parr correlation functional, denoted by B3LYP, were employed in the calculations using density functional theory (DFT). The DFT calculations at B3LYP/6-31G (d,p) and B3LYP/311+G (d,p) levels, MP2 calculations, and the density functional from Truhlar group (hybrid GGA: MPW1k) [69–71] were carried out using the quantum chemical package Gaussian-2009 [72]. Calculations were carried out based on the restricted Hartree-Fock method [72]. The starting geometries of all calculated molecules were obtained using the Argus Lab program [73] and were initially optimized at the HF/6-31G level of theory, followed by optimization at the B3LYP/6-31G (d,p), B3LYP/6-311+G (d,p), MPW1k (mpwpw91/6-31+G (d,p)), or MP2/6-31G (d,p) levels. Total geometry optimizations included all internal rotations. Second derivatives were estimated for all 3N-6 geometrical parameters during optimization. In the DFT calculations for 1–9 and atenolol ProD 1-2, two types of conformations in particular were considered: one in which the amide carbonyl is *syn* to the carboxyl group and another in which it is *anti*. An energy minimum (a stable compound or a reactive intermediate) has no negative vibrational force constant. A transition state is a saddle point which has only one negative vibrational force constant [74]. Transition states were located first by the normal reaction coordinate method [75] where the enthalpy changes were monitored by stepwise changing the interatomic distance between two specific atoms. The geometry at the highest point on the energy profile was reoptimized by using the energy gradient method at the B3LYP/6-31G (d,p) level of theory [75]. The “reaction coordinate method” was used to calculate the activation energy in maleamic (4-amino-4-oxo-2butenoic) acids (Kirby's N-alkylmaleamic acids), 1–9, and atenolol ProD 1-2. In this method, one bond length is constrained for the appropriate degree of freedom while all other variables are freely optimized. The activation energy values for the approach and dissociation steps were calculated from the difference in energies of the global minimum structures (GM) and the derived transition states. Verification of the desired reactants and products was accomplished using the “intrinsic coordinate method” [75]. The transition state structures were verified by their only one negative frequency. Full optimization of the transition states was accomplished after removing any constrains imposed while executing the energy profile. The activation energies obtained from DFT at B3LYP/6-31G (d,p) level of theory for 1–9 and atenolol ProD 1-2 were calculated with and without the inclusion of solvent (water and ether). The calculations with the incorporation of a solvent were performed using the integral equation formalism model of the Polarizable Continuum Model (PCM) [76–79].

## 3. Results and Discussion

**Figure 2:**
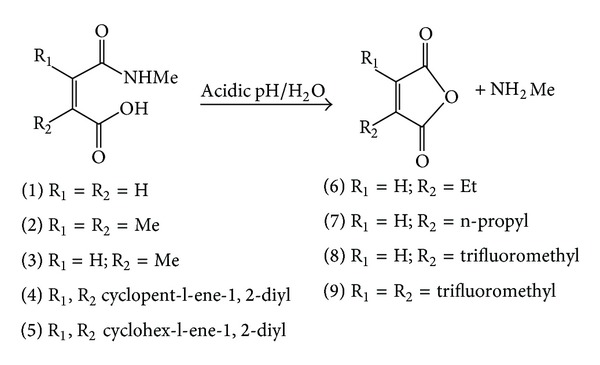
Acid-catalyzed hydrolysis for N-alkylmaleamic acids 1–9.

**Figure 3:**
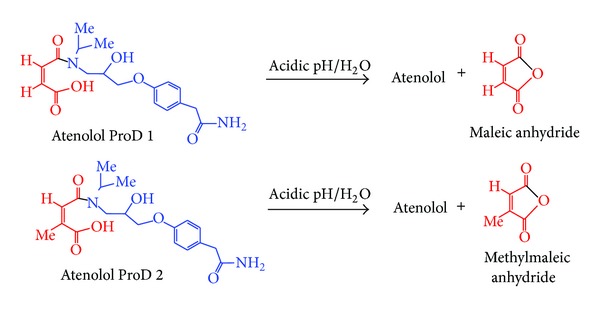
Acid-catalyzed hydrolysis for atenolol ProD 1-ProD 2.

Our proposed atenolol prodrugs that were designed based on the acid-catalyzed hydrolysis reactions of N-alkyl maleamic acids 1–9 (Figure 2) are depicted in Figure 3. As illustrated in Figure 3, the only difference between the proposed atenolol prodrugs and the parent drug, atenolol, is that the amine group in atenolol was replaced with an amide moiety. This chemical change is expected to increase the stability of the alcohol derivative which is formed (prodrug) compared to the corresponding amine alcohol, atenolol, due to general chemical stability for tertiary alcohols over amine alcohols. In addition, stability studies on atenolol ester derivatives have showed the ester derivatives to be much more stable than their corresponding alcohol, atenolol, upon formulating in aqueous solutions. On the other hand, kinetic study of atenolol and propranolol revealed that increasing the lipophilicity of the drug leads to an increase in the stability of its aqueous solutions. Based on that, it is expected that atenolol prodrugs (atenolol amide derivatives) shown in Figure 3 will be more resistant to heat or/oxidation when standing in aqueous solutions [7, 11, 80–83].

The drug's bitter taste can be masked by utilizing the prodrug chemical approach. For example, paracetamol, a widely used pain killer and fever reducer found in the urine of patients who had taken phenacetin, has a very unpleasant bitter taste [84]. Phenacetin, on the other hand, lacks or has very slight bitterness [85]. The difference in the structural features of both drugs is only in the nature of the group in the *para* position of the benzene ring. While in the case of paracetamol the group is hydroxyl, in phenacetin, it is ethoxy. Acetanilide has a chemical structure similar to that of paracetamol and phenacetin but lacks the group in the *para* position of the benzene ring, making it lack the bitter taste characteristic of paracetamol [86]. These combined facts suggest that the presence of the hydroxy group on the *para* position is the major contributor for the bitter taste of paracetamol. It is believed that paracetamol interacts with the bitter taste receptors *via* hydrogen bonding which involves its phenolic group. In fact, our recent study on binding of paracetamol and its prodrugs to bitter taste receptors has confirmed that prodrugs lacking the hydroxyl group have no bitterness. Similarly, it is expected that blocking the amine group in atenolol with a suitable linker might inhibit the interaction between the amine group in atenolol and its bitter taste receptors and hence masks its bitterness. The nature of the bitter taste receptors with either paracetamol (via the phenolic group) or atenolol (via the amine group) is likely to be as a result of hydrogen bonding between the substrate and the receptor [25].

As shown in Figure 3, the proposed atenolol prodrugs, atenolol ProD 1 and atenolol ProD 2, have a hydroxyl and carboxylic acid groups (hydrophilic moiety) and the rest of the prodrug molecule is a lipophilic moiety, where the combination of both groups ensures a molecule with a moderate hydrophilic lipophilic balance (HLB).

It is worthy to note that the HLB value of atenolol prodrug moiety will be largely determined on the medium (physiologic environment) by which the prodrug is dissolved. For instance, in the stomach (pH 1-2), atenolol prodrugs will exist in the free carboxylic acid form, whereas in the blood circulation (pH 7.4) the carboxylate anion form will be dominant. It is planned that atenolol ProD 1-ProD 2 (Figure 3) will be formulated as sodium carboxylate salt since this form is expected to be stable in neutral aqueous medium. However, upon exposure to stomach (pH less than 3), the prodrugs will exist mainly as free carboxylic acid forms thus enabling the acid-catalyzed hydrolysis to proceed.

### 3.1. Atenolol Prodrugs Design

#### 3.1.1. DFT Mechanistic Study of the Acid-Catalyzed Hydrolysis of 1–9, Atenolol ProD 1, and Atenolol ProD 2

**Figure 4:**
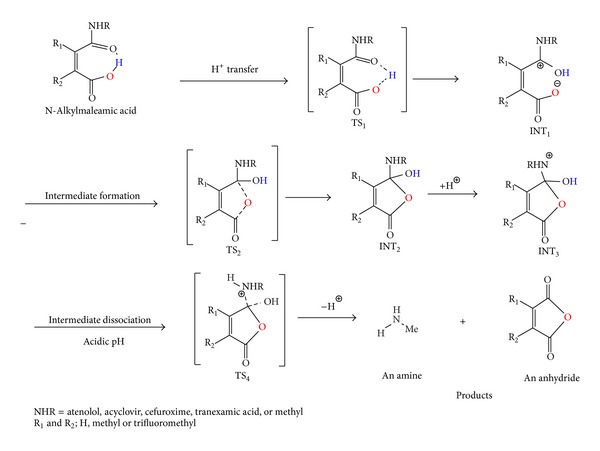
Mechanistic pathway for the acid-catalyzed hydrolysis of 1–9 and atenolol ProD 1-ProD 2. TS and INT are tetrahedral intermediate and transition state, respectively.

The acid-catalyzed hydrolysis of 1–9 (Figure 2) was extensively studied by Kirby and Lancaster. The study findings demonstrate that the amide bond cleavage is due to intramolecular nucleophilic catalysis by the adjacent carboxylic acid group and the rate-limiting step is the dissociation of the tetrahedral intermediate (Figure 4) [38]. Two decades later, the reaction was theoretically investigated by Katagi using AM1 semiempirical calculations. In contrast to what was proposed by Kirby, the AM1 study revealed that the rate-limiting step is the tetrahedral intermediate formation and not its collapse [87]. Later on, Kluger and Chin have studied the intramolecular hydrolysis mechanism of a number of N-alkylmaleamic acids derived from aliphatic amines having a wide range of basicity [88]. Their findings revealed that the identity of the rate-limiting step is a function of both the basicity of the leaving group and the acidity of the solution.

Based on the above-mentioned experimental findings we believe that replacing the N-methylamide group in 1–9 (Figure 2) with atenolol molecule, as shown for atenolol ProD 1 and ProD 2 in Figure 3, will not have any significant effect on the relative rates of these processes, if they proceed in the same mechanism. Therefore, DFT calculations of the kinetic and thermodynamic properties for processes 1–9 might fairly predict the rates for the chemical intraconversion of atenolol ProD 1 and ProD 2.

#### 3.1.2. The Factors Determining the Reaction Rate-Limiting Step

Our previous calculations of the mechanism for the acid-catalyzed hydrolysis of 1–7 demonstrate that the process involves at first a proton transfer from the carboxylic group into the adjacent amide carbonyl carbon followed by a nucleophilic attack of the carboxylate anion thus formed onto the protonated carbonyl carbon to form a tetrahedral intermediate which in further step dissociates to give products (Figure 4).

**Figure 5:**
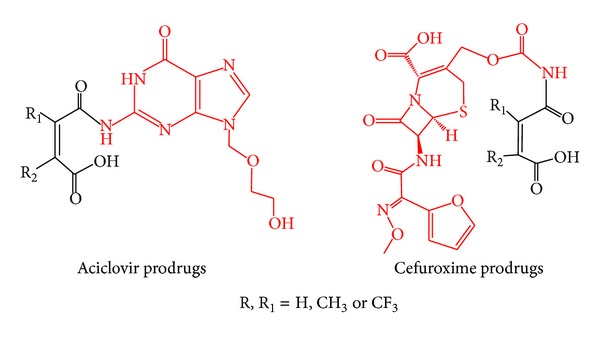
Chemical structures for aciclovir and cefuroxime prodrugs.

In addition, the DFT calculation results were found to be in accordance with the reports by Kirby and Lancaster. [38] and Kluger and Chin [88] that indicate that the N-alkylmaleamic acids hydrolysis occurs by one mechanism where the rate-limiting step is largely dependent on the nature of the amine leaving group and the medium solvent. Two different rate-limiting steps were suggested: (1) formation of atetrahedral intermediate [87] and (2) a tetrahedral intermediate dissociation (Figure 4) [38, 88]. Our gas phase DFT calculated barriers revealed that the rate-limiting step for the hydrolysis of all maleamic acid amides studied regardless of the nature of the amine leaving group is a tetrahedral formation, whereas when the calculations were run in a dielectric constant of 78.39 (water), the rate-limiting step for the hydrolysis of acid amides with primary amine was the dissociation of the tetrahedral intermediate; however, when an acyclovir (antiviral drug) or a cefuroxime (antibacterial drug) moieties (for prodrug structures see Figure 5) were a leaving group, the tetrahedral intermediate formation was found to be involved in the hydrolysis rate-limiting step.

In order to assign the factors creating the unusual accelerations in rate and to explore whether the discrepancy in processes 1–9 rates stems from strain energy or other effects using Allinger's MM2 method [36], the strain energies for the reactants (GM), intermediates (INT), and products (P) in 1–9 and atenolol ProD 1-2 were calculated and their values were examined for correlation with the corresponding experimental relative rates, log*k*_*rel*_. Strong correlations were obtained with a correlation coefficient *R* = 0.94–0.97. Similarly, fair correlations were obtained between the experimental and calculated free activation energy values and strain energy values of the intermediates and reactants (E_s_ INT-GM) (*R* = 0.85–0.94). The combined correlation results imply that the rate of the reaction for systems having less strained intermediates or products such as 2 and 5 are higher than that having more strained intermediates or products such as 1 and 4. This might be attributed to the fact that the transition state structures resemble that of the corresponding intermediate.

#### 3.1.3. Calculation of the *t*_1/2_ Values for the Cleavage Reactions of Prodrugs ProD 1-2

Intramolecular efficiency is widely accepted to be measured by its effective molarity (EM) value. EM is defined as a ratio of the intramolecular and its corresponding intermolecular rate, where both processes are driven by identical mechanisms. The major factors affecting the EM parameter are the size of a ring, reaction medium, and reaction type. Cyclization processes which proceed* via *intramolecular nucleophilic addition are much more efficient than intramolecular proton transfers. Values in the order of 10^9^–10^13^ M have been reported for the EM parameter in intramolecular processes occurring through nucleophilic addition. On the other hand, EM values less than 10 M were measured for proton transfer processes until recently where values of 10^10^ were reported by Kirby on the hydrolysis of some enzyme models [40–46, 89–92].

For obtaining credibility to our calculation results, we introduce our computation rational for calculating the EM values for processes 1–9 and ProD 1-2 based on the DFT calculated activation energies (Δ*G* ^‡^) of 1–9 and ProD 1-2, and the corresponding intermolecular process Inter (Figure 5 and Table 1).

Using (1)–(4), (5) which describes the EM parameter as a function of the difference in the activation energies of the intramolecular and the corresponding intermolecular processes was derived. The calculated EM values for processes 1–9 and ProD 1-2 were obtained as

(1) $$\begin{array}{c} \Delta G_{\text{inter}}^{‡}=-RT\ln k_{\text{inter}}, \end{array}$$

(2) $$\begin{array}{c} \Delta G_{\text{intra}}^{‡}=-RT\ln k_{\text{intra}}, \end{array}$$

(3) $$\begin{array}{c} \text{EM}=\frac{k_{\text{intra}}}{k_{\text{inter}}}, \end{array}$$

(4) $$\begin{array}{c} {\Delta G}_{\text{intra}}^{‡}-{\Delta G}_{\text{inter}}^{‡}=-RT\ln\frac{k_{\text{intra}}}{k_{\text{inter}}}, \end{array}$$

(5) $$\begin{array}{c} \text{EM}=e^{-({\Delta G}_{\text{intra}}^{‡}\;-\;\Delta G_{\text{inter}}^{‡})/RT}, \end{array}$$

where *T* is the temperature in Kelvin and *R* is the gas constant.

**Figure 6:**
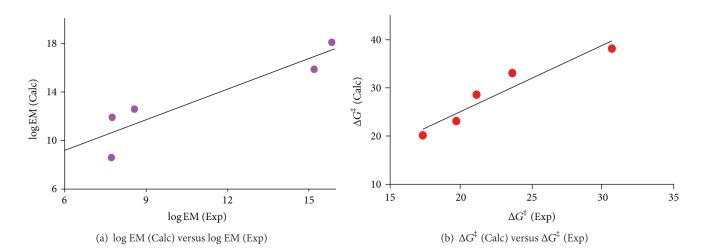
(a) Log calculated effective molarity versus experimental effective molarity for processes 1–5. (b) DFT calculated activation energy (kcal/mol) versus experimental activation energy (kcal/mol) for processes 1–5.

The calculated log EM values for 1–5 were examined for correlation with the log EM experimental values [38] (Figure 6(a)). The correlation results revealed that 2 and 5 were the most efficient processes among 1–5, whereas process 4 was the least. The discrepancy in rates between 2 and 5 on one hand and 4 on the other hand is attributed to strain effects.

Figure 6(a) indicates that although the calculated and experimental EM values are comparable, their absolute values slightly differ. This might be due to the fact that the experimental EM values for 1–5 were measured in the presence of aqueous acid, whereas the DFT calculations were run in plain water. The dielectric constant value for a mixture of acid/water is expected to be different from pure water (78.39) and hence the discrepancy in the calculated and experimental EM values.

In addition, for further support to the credibility of our DFT calculations, the calculated free activation energies in water (Δ*G*_BW_^‡^) were correlated with the experimental free activation energies (ExpΔ*G* ^‡^). Strong correlation was obtained with *R* value of 0.96 (Figure 6(b)).

Using (6) obtained from the correlation of log *k*_*rel*_ versus Δ*G* ^‡^ and the *t*_1/2_ value for process 2 (*t*_1/2_ = 1 second)^38^, the *t*_1/2_ values for ProD 1 and ProD 2 at pH 2 were calculated and their values were 65.3 hours and 11.8 minutes, respectively. Consider

(6) $$\begin{array}{c} \log k_{\mathrm{rel}}=-0.44\;\Delta G^{‡}+13.53. \end{array}$$

#### 3.1.4. Hydrolysis Studies

**Figure 7:**
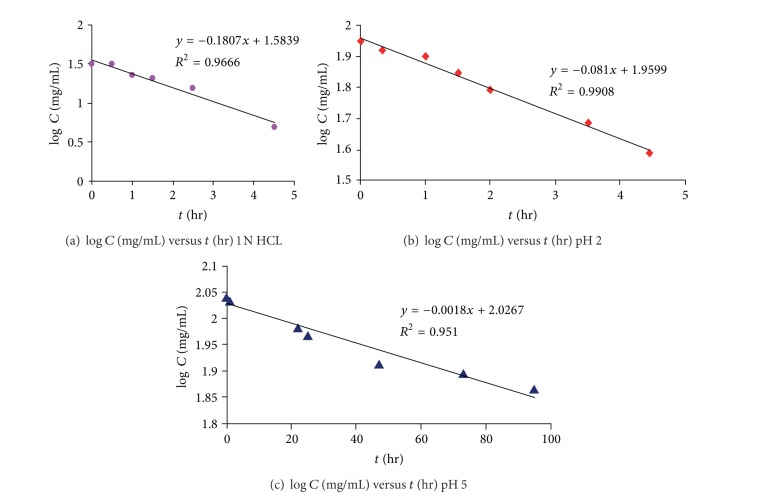
First-order hydrolysis plot of atenolol ProD 1 in (a) 1 N HCl, (b) buffer pH 2, and (c) buffer pH 5.

The kinetics of the acid-catalyzed hydrolysis study for atenolol ProD 1 was carried out in an aqueous buffer in a similar manner to that done by Kirby on N-alkylmaleamic acids 1–7. This is for investigating whether atenolol prodrug hydrolyzes in aqueous medium and to what extent, suggesting its fate in the system. Acid-catalyzed hydrolysis kinetics of the synthesized atenolol ProD 1 was studied in four different aqueous media: 1 N HCl and buffers pH 2, pH 5, and pH 7.4. Under the experimental conditions, the target prodrug (atenolol ProD 1) was hydrolyzed to release the parent drug, atenolol, (Figure 7) as was evident by HPLC determination. At constant pH and temperature, the reaction displayed strict first-order kinetics as the *k*_obs_ was fairly constant and a straight line was obtained on plotting log concentration of residual prodrug versus time. The rate constant (*k*_obs_) and the corresponding half-lives (*t*_1/2_) for atenolol prodrug ProD 1 in the different media were calculated from the linear regression equation correlating the log concentration of the residual prodrug versus time. The kinetic data, *k*_obs_ and *t*_1/2_ values, are listed in Table 2. One N HCl, pH 2, and pH 5 were selected to examine the interconversion of atenolol ProD 1 in pH as of stomach, because the mean fasting stomach pH of adult is approximately 1-2 and increases up to 5 following ingestion of food. In addition, buffer pH 5 mimics the beginning of the small intestine pathway. The medium at pH 7.4 was selected to examine the interconversion of the tested atenolol prodrug in the blood circulation system. Acid-catalyzed hydrolysis of the atenolol ProD 1 was found to be higher in 1 N HCl than at pH 2 and 5 (Figure 7). At 1 N HCl, the atenolol ProD 1 was hydrolyzed to release the parent drug in 2.53 hours. On the other hand, at pH 7.4, the prodrug was entirely stable and no release of the parent drug was observed. Since the p*K*_a_ of atenolol ProD 1 carboxylic acid is in the range of 3-4, it is expected at pH 5 that the anionic form of the prodrug will be dominant and the percentage of the free acidic form that undergoes the acid-catalyzed hydrolysis will be relatively low. At 1 N HCl and pH 2, most of the prodrug will exist as the free acid form, whereas at pH 7.4 most of the prodrug will be in the anionic form. Thus, the difference in rates at the different pH buffers.

## 4. Conclusions

The QM calculations at different levels demonstrated that the efficiency of Kirby's N-alkylmaleamic acids and atenolol ProD 1-ProD 2 is largely sensitive to the pattern of substitution on the carbon-carbon double bond and nature of the alkyl group on amide nitrogen. The linear correlation found between the acid-catalyzed hydrolysis strain energy difference between the intermediate and the reactant (E_s_ INT-GM) suggests that the reaction is governed by strain effect. Moreover, a strong correlation obtained from plotting the calculated and experimental EM values (effective molarity) reinforces the credibility of using such methods for energy and rate predictions of the kind of processes reported herein.

**Figure 8:**
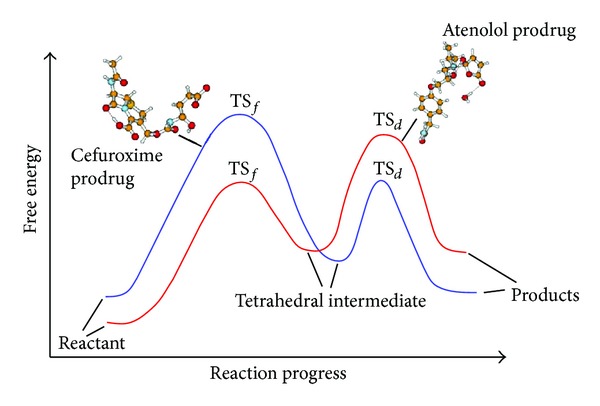
Energy profiles for the acid-catalyzed hydrolysis of atenolol and cefuroxime prodrugs.

Comparison of the calculated DFT properties for 1–7 atenolol prodrugs ProD 1-ProD 2 with previously calculated properties for the acid-catalyzed hydrolysis of acyclovir prodrugs and cefuroxime ProD 1–ProD 4 (Figure 5) indicates that while, for systems 1–7 and atenolol prodrugs ProD 1-ProD 2, the rate-limiting step was the breakdown of the tetrahedral intermediate in the reactions of cefuroxime prodrugs ProD 1–ProD 4 and acyclovir prodrugs ProD 1–ProD 4, the rate-limiting step was the formation of the tetrahedral intermediate (Figure 8). This might be due to the nature of the amine leaving group involved in the tetrahedral intermediate dissociation step.

Using the correlation equation obtained from the plot of the calculated and experimental EM values, the *t*_1/2_ values of two different atenolol prodrugs (ProD 1-ProD 2) were estimated.

Comparison between the calculated *t*_1/2_ values 63.2 hours) for atenolol ProD 1 to the experimental value (3.82 hours) indicates that while the value obtained by B3LYP/6-31G (d,p) is overestimated (about 17 times larger than the experimental), the values obtained by mpwpw91/6-31+G (d,p) were much more closer, 6.3 hours. This discrepancy between the calculated and experimental values might be attributed to the following. (1) B3LYP/6-31 G (d,p) is a DFT method without dispersion corrections and (2) PCM solvation model (calculations in presence of solvent) is not capable of handling calculations in acidic aqueous solvent (medium), since the dielectric constant for pH 2 aqueous solutions is not known. In the study, calculations of the value of 78.39 (dielectric constant for pure water) were used instead (it should be noted that DFT calculations in the presence of a mixture of acid and water are not feasible).

The *t*_1/2_ experimental value at pH 5 was 133 hours and at pH 7.4 no interconversion was observed. The lack of the reaction at the latter pH might be due to the fact that at this pH atenolol ProD 1 exists solely in the ionized form (p*K*_a_ about 3-4). As mentioned before, the free acid form is a mandatory requirement for the reaction to proceed.

Future strategy to achieve more efficient atenolol prodrugs capable of increasing the liquid formulation stability, eliminating atenolol bitterness, and releasing the parent drug in a programmable manner is (a) synthesis of atenolol prodrugs having p*K*_a_ around 6 (intestine pH) such as atenolol ProD 3; (ii) *in vitro* kinetic studies of atenolol ProD 3 performed at pH 6.5 (intestine) and pH 7.4 (blood circulation system); and (iii) *in vivo* pharmacokinetic studies done in order to determine the bioavailability and the duration of action of the tested prodrug. Furthermore, based on the *in vivo* pharmacokinetics characteristics of atenolol ProD 3, new prodrugs may be design and synthesized.

## Figures and Tables

**Table 1:** Experimental and DFT calculated kinetic and thermodynamic properties for the acid-catalyzed hydrolysis of 1–9, Inter, and atenolol ProD 1-ProD 2.

System	B3L Δ*H* ^‡^_d_ (kcal/mol)	B3L311 Δ*H* ^‡^_d_ (kcal/mol)	MPW1k Δ*H* ^‡^_d_ (kcal/mol)	MP2 Δ*H* ^‡^_d_ (kcal/mol)	log EM (Exp)	log EM (Calc)	Exp Δ*G* ^‡38^ (kcal/mol)
(1)	27.31	27.42	25.07	25.75	7.724	8.52	23.70
(2)	13.93	14.02	11.96	10.41	15.86	18.08	17.30
(3)	24.41	23.85	22.61	21.51	7.742	11.93	21.14
(4)	34.42	—	—	—	1.255	4.81	30.70
(5)	13.25	—	—	—	15.190	15.82	19.75
(6)	23.83	—	—	—	6.962	12.76	—
(7)	24.86	—	—	—	8.568	12.57	—
(8)	24.08	—	—	—	—	6.36	
(9)	17.88	—	—	—	—	21.68	
Atenolol ProD 1	20.08	—		—	—	4.06	
Atenolol ProD 2	15.43	—	—	—	—	13.11	
Inter	39.90	—	—	—	—	—	—

B3LYP refers to values calculated by B3LYP/6-31G (d,p) method. Δ*H* ^‡^ is the calculated activation enthalpic energy (kcal/mol). *T*Δ*S* ^‡^ is the calculated activation entropic energy (kcal/mol). Δ*G* ^‡^ is the calculated activation free energy (kcal/mol). EM: *e* ^−(Δ*G*‡inter−Δ*G*‡intra)/RT^. BW refers to tetrahedral intermediate breakdown calculated in water. Exp refers to experimental value. Calc refers to DFT calculated values.

**Table 2:** First-order hydrolysis plot of atenolol ProD 1 in (a) 1N HCl, (b) buffer pH 2, and (c) buffer pH 5.

Medium	*k*_obs_ (hours^−1^)	*t*_1/2_ (hours)
1N HCl	4.95 × 10^−4^	2.53
Buffer pH 2	2.22 × 10^−4^	3.82
Buffer pH 5	2.75 × 10^−6^	133
Buffer pH 7.4	—	—
